# Bis[(methyl­sulfan­yl)carbon­yl]disulfane

**DOI:** 10.1107/S1600536812024750

**Published:** 2012-06-13

**Authors:** David K. Ford, Victor G. Young Jr, George Barany

**Affiliations:** a Department of Chemistry, University of Minnesota, Minneapolis, MN 55455, USA

## Abstract

The title compound, C_4_H_6_O_2_S_4_, was prepared by repeating, with subtle improvements, a multi-step route originally described by Mott & Barany [*J. Chem. Soc. Perkin Trans. 1* (1984) ▶, pp. 2615–2621]. The title compound was obtained for the first time as a crystalline material. The two [(methyl­sulfan­yl)carbon­yl]sulfenyl moieties are essentially perpendic­ular to each other, each approximately planar (r.m.s. deviations of 0.02 and 0.01 Å) and with a C—S—S—C torsion angle = 90.99 (6)°, which compares well with the theoretical value of 90°.

## Related literature

For the preparation of the title compound and for the preparation and structures of the corresponding trisulfane and tetrasulfane compounds, see: Mott & Barany (1984 ▶). For other related structures, see: Bereman *et al.* (1983 ▶); Rout *et al.* (1983 ▶); Paul & Srikrishnan (2004 ▶); Li *et al.* (2006 ▶); Schroll *et al.* (2012 ▶). For a description of the Cambridge Structural Database, see: Allen (2002 ▶). For optimum dihedral angles, see: Pauling (1960 ▶). For background to isomeric bis­(alk­oxy­thio­carbon­yl)­poly­sulfanes, see: Reid (1962 ▶). 
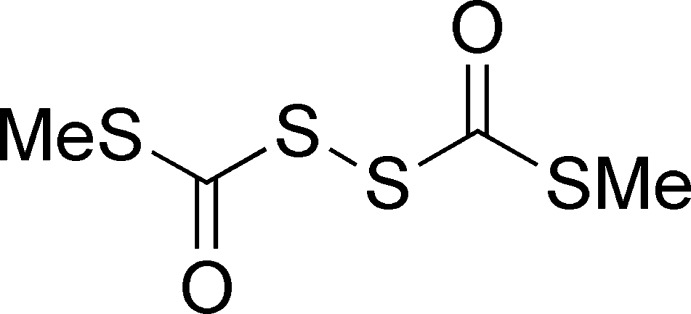


## Experimental

### 

#### Crystal data

C_4_H_6_O_2_S_4_
*M* *_r_* = 214.33
Triclinic, 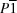

*a* = 5.3300 (7) Å
*b* = 8.6935 (12) Å
*c* = 9.9166 (13) Å
α = 109.875 (2)°
β = 92.154 (2)°
γ = 101.481 (2)°
*V* = 420.71 (10) Å^3^
*Z* = 2
Mo *K*α radiation
μ = 1.07 mm^−1^
*T* = 123 K
0.35 × 0.30 × 0.25 mm

#### Data collection

Bruker SMART CCD area-detector diffractometer
Absorption correction: multi-scan (*SADABS*; Sheldrick, 2008*a* ▶) *T*_min_ = 0.707, *T*_max_ = 0.776
5024 measured reflections
1894 independent reflections
1774 reflections with *I* > 2σ(*I*)
*R*_int_ = 0.022

#### Refinement

*R*[*F* ^2^ > 2σ(*F* ^2^)] = 0.021
*wR*(*F* ^2^) = 0.054
*S* = 1.07
1894 reflections
93 parameters
H-atom parameters constrained
Δρ_max_ = 0.36 e Å^−3^
Δρ_min_ = −0.24 e Å^−3^

### 

Data collection: *SMART* (Bruker, 1998 ▶); cell refinement: *SMART*; data reduction: *SAINT* (Bruker, 1998 ▶); program(s) used to solve structure: *SHELXTL* (Sheldrick, 2008*b* ▶); program(s) used to refine structure: *SHELXTL*; molecular graphics: *SHELXTL*; software used to prepare material for publication: *SHELXTL*.

## Supplementary Material

Crystal structure: contains datablock(s) I, global. DOI: 10.1107/S1600536812024750/gw2119sup1.cif

Structure factors: contains datablock(s) I. DOI: 10.1107/S1600536812024750/gw2119Isup2.hkl

Supplementary material file. DOI: 10.1107/S1600536812024750/gw2119Isup3.cml

Additional supplementary materials:  crystallographic information; 3D view; checkCIF report

## Figures and Tables

**Fig. 1.**
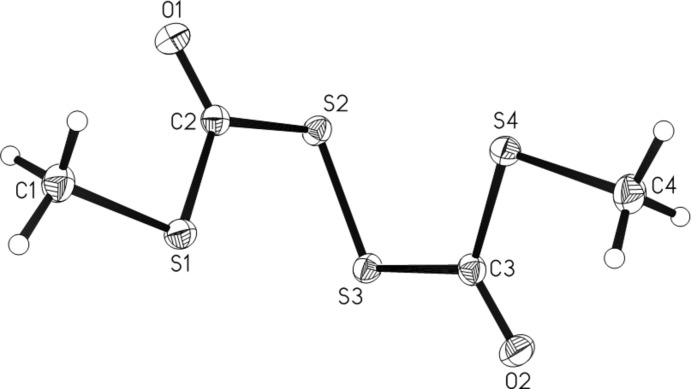
Crystallographic structure of bis[(methylsulfanyl)carbonyl]disulfane with all non-hydrogen atoms labeled and numbered.

**Fig. 2.**
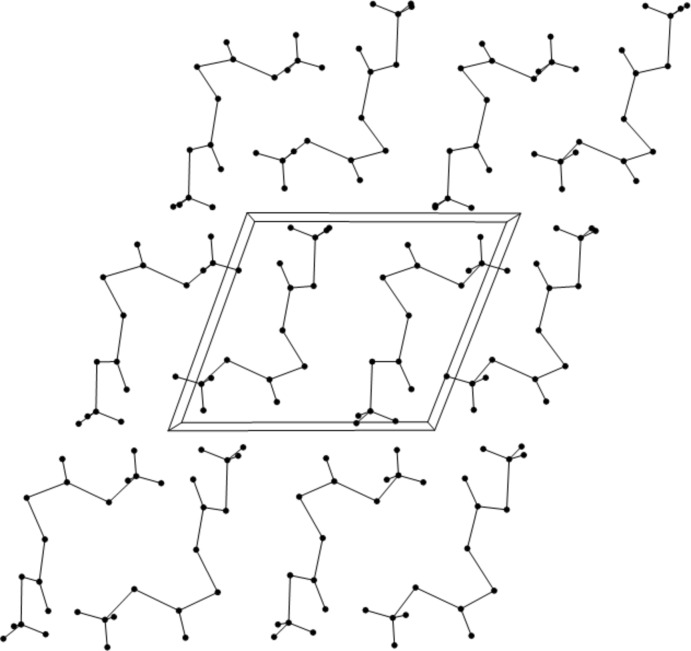
Packing diagram for bis[(methylsulfanyl)carbonyl]disulfane.

**Fig. 3.**
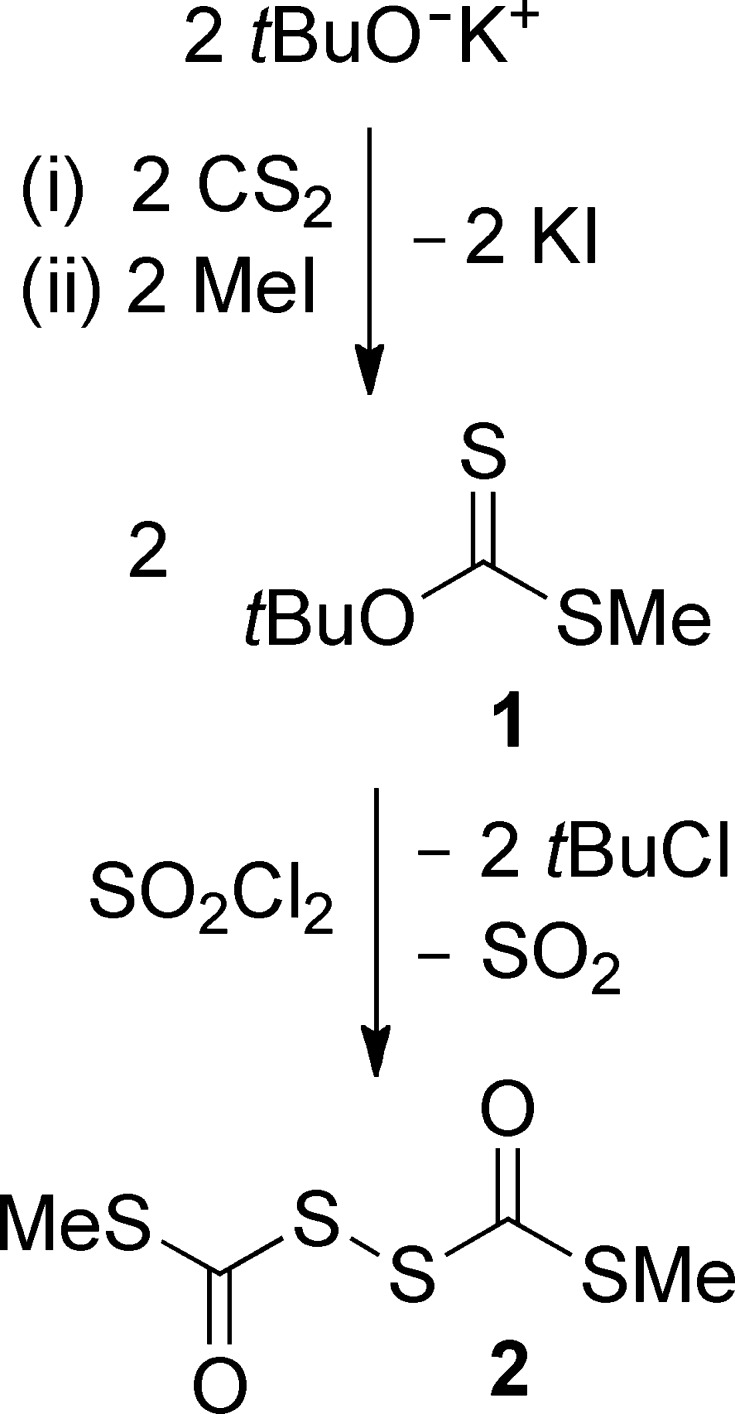
Chemistry used to prepare bis[(methylsulfanyl)carbonyl]disulfane (experimental procedures are provided herein).

## supplementary crystallographic information

### Comment

While bis(alkoxysulfanylcarbonyl)polysulfanes, [*R*O(C═S)]_2_S*_n_* (*n* = 1, 2, 3, 4) have been known for a long time due to their derivation from readily formed xanthate salts (Reid, 1962), much less information is available about the isomeric bis[alkyl(sulfanylcarbonyl)]polysulfanes, [*R*S(C═O)]_2_S*_n_* (Mott & Barany, 1984). In 1984, we reported methodology for the preparation of reasonably pure (>95%) exemplars in the latter family for *R* = Me; the trisulfane and tetrasulfane of the series were obtained as crystals and their structures were solved by X-ray diffraction (Mott & Barany, 1984). Beyond the successful synthetic routes reported therein, a number of alternative methods were tested, which seemed rather straightforward and were well precedented for analogous compounds, but failed for the series under investigation. For the present studies, we carried out more careful experimental work aimed at the disulfane (Fig. 3), and obtained it in crystalline form for the first time. The structure of the disulfane was solved by X-ray crystallography, and compared to the structures determined earlier.

All bond distances and angles are within expected ranges. Because the disulfane is adjacent to carbonyl groups, the S—S bond length of 2.03 Å is slightly shorter than the 2.07 Å reported for the S—S bond length in S_8_. This phenomenon is quite general and has been observed for related compounds. In all, a search of the Cambridge Database for compounds of the formula *R*(C═O)SS(C═O)*R* provided four different compounds for comparison [CSD refcodes: BOWGAV (Bereman *et al.*, 1983), DBZOSS01&03 (Rout *et al.*, 1983; Paul & Srikrishnan, 2004), UDALER (Li *et al.*, 2006), and 880326 (Schroll *et al.*, 2012)]. The most noteworthy feature of our newly reported structure is that the torsion angle about the disulfane was 90.99°, which comes closer to the theoretical optimum of 90° (Pauling, 1960) than any other of the comparison compounds with the exception of bis(*N*,*N*-dicyclohexylsulfanylcarbamoyl)disulfane, where this angle is 89.7° (Li *et al.*, 2006).

*Note regarding nomenclature:* The title compound is named in a manner that is consistent with our prior publications and modern conventions. The related compound studied by Li *et al.* (2006) was named bis(*N*,*N*-dicyclohexylsulfanylcarbamoyl)disulfide by those authors.

### Experimental

*S*-Methyl*O*-*t*-butyl disulfanylcarbonate (1). First, potassium *t*-butyl xanthate was prepared by adding carbon disulfide (37.0 ml, 0.61 mol) over 15 min to a well stirred suspension of potassium *t*-butoxide (69.7 g, 0.62 mol) in *p*-xylene (450 ml) at 80°C. The reaction mixture was allowed to cool to 25°C, and the orange-yellow precipitate which had formed was collected on a Buchner funnel, and washed with ethyl ether (1.7 l total, gravity filtration) over a 1 h period. After the final ether wash, the solid was air-dried by suction applied to the filtration apparatus from a water aspirator, and then dried further in a desiccator (20 mm) for 72 h (time required to achieve constant mass). Yield of xanthate salt: 80.3 g (70%). A portion of the xanthate salt (23.9 g, 127 mmol) was suspended in ether (200 ml), and neat iodomethane (12.0 ml, 193 mmol) was added, under magnetic stirring, at 25°C over a 2 min period. After 24 h, the reaction mixture was filtered and concentrated *in vacuo* to provide an unstable yellow oil (10.0 g, 48%) that was stored at -20°C and was usable for about 4 months. ^1^H NMR (CDCl_3_, 300 MHz) δ 2.46 [s, 3H, SMe], 1.70 [s, 9H, *t*BuO].

Bis[(methylsulfanyl)carbonyl]disulfane (2)*.* Neat sulfuryl chloride (1.3 ml, 16.2 mmol) was added, with stirring at 4°C, over 1 min, to a solution of compound 1 (5.0 g, 30.4 mmol) in CHCl_3_ (20 ml). After completion of addition, stirring continued for a further 5 min, following which the homogeneous reaction mixture was concentrated *in vacuo* to provide the crude title product as a yellow oil [3.4 g, nominally quantitative, but comprising primarily desired 2 and starting 1 in a molar ratio of 7:4, along with smaller amounts of other impurities that were not identified further]. A portion (2.0 g) of the crude oil was purified by silica gel chromatography, eluted with hexanes–ethyl acetate (6:1). The purest fractions were placed under petroleum ether at -20°C, whereupon white crystals (0.27 g, >99% pure, 14% yield), m.p. 31–32°C, formed within 24 h. ^1^H NMR (CDCl_3_, 300 MHz) δ 2.48 [s, 6H, SCH_3_].

### Refinement

All of the H atoms were positioned geometrically (C—H = 0.96 Å) and refined as riding with *U*_iso_(H) = 1.2*U*_eq_(C).

### Crystal data

**Table:**

C_4_H_6_O_2_S_4_	*Z* = 2
*M**_r_* = 214.33	*F*(000) = 220
Triclinic, *P*1	*D*_x_ = 1.692 Mg m^−^^3^
*a* = 5.3300 (7) Å	Mo *K*α radiation, λ = 0.71073 Å
*b* = 8.6935 (12) Å	Cell parameters from 2965 reflections
*c* = 9.9166 (13) Å	θ = 2.2–27.5°
α = 109.875 (2)°	µ = 1.07 mm^−^^1^
β = 92.154 (2)°	*T* = 123 K
γ = 101.481 (2)°	Block, colourless
*V* = 420.71 (10) Å^3^	0.35 × 0.30 × 0.25 mm

### Data collection

**Table:**

Bruker SMART CCD area-detector diffractometer	1894 independent reflections
Radiation source: sealed tube	1774 reflections with *I* > 2σ(*I*)
Graphite monochromator	*R*_int_ = 0.022
φ and ω scans	θ_max_ = 27.5°, θ_min_ = 2.2°
Absorption correction: multi-scan (*SADABS*; Sheldrick, 2008*a*)	*h* = −6→6
*T*_min_ = 0.707, *T*_max_ = 0.776	*k* = −11→10
5024 measured reflections	*l* = 0→12

### Refinement

**Table:**

Refinement on *F*^2^	Primary atom site location: structure-invariant direct methods
Least-squares matrix: full	Secondary atom site location: difference Fourier map
*R*[*F*^2^ > 2σ(*F*^2^)] = 0.021	Hydrogen site location: inferred from neighbouring sites
*wR*(*F*^2^) = 0.054	H-atom parameters constrained
*S* = 1.07	*w* = 1/[σ^2^(*F*_o_^2^) + (0.0267*P*)^2^ + 0.1517*P*] where *P* = (*F*_o_^2^ + 2*F*_c_^2^)/3
1894 reflections	(Δ/σ)_max_ = 0.001
93 parameters	Δρ_max_ = 0.36 e Å^−^^3^
0 restraints	Δρ_min_ = −0.24 e Å^−^^3^

### Special details

**Table:**

Geometry. All e.s.d.'s (except the e.s.d. in the dihedral angle between two l.s. planes) are estimated using the full covariance matrix. The cell e.s.d.'s are taken into account individually in the estimation of e.s.d.'s in distances, angles and torsion angles; correlations between e.s.d.'s in cell parameters are only used when they are defined by crystal symmetry. An approximate (isotropic) treatment of cell e.s.d.'s is used for estimating e.s.d.'s involving l.s. planes.
Refinement. Refinement of *F*^2^ against all reflections. The weighted *R*-factor *wR* and goodness of fit *S* are based on *F*^2^, conventional *R*-factors *R* are based on *F*, with *F* set to zero for negative *F*^2^. The threshold expression of *F*^2^ > 2σ(*F*^2^) is used only for calculating *R*-factors(gt) *etc*. and is not relevant to the choice of reflections for refinement. *R*-factors based on *F*^2^ are statistically about twice as large as those based on *F*, and *R*-factors based on all data will be even larger.

### Fractional atomic coordinates and isotropic or equivalent isotropic displacement parameters (Å^2^)

**Table:**

	*x*	*y*	*z*	*U*_iso_*/*U*_eq_	
S1	0.14601 (6)	0.32879 (4)	0.88175 (3)	0.01738 (9)	
S2	0.29712 (6)	0.29567 (4)	0.58244 (3)	0.01738 (9)	
S3	0.59396 (6)	0.46586 (4)	0.72293 (4)	0.01720 (9)	
S4	0.23910 (6)	0.67401 (4)	0.66158 (4)	0.01664 (9)	
O1	−0.13318 (18)	0.13895 (12)	0.63241 (11)	0.0206 (2)	
O2	0.68271 (19)	0.78866 (12)	0.83115 (12)	0.0242 (2)	
C1	−0.1386 (3)	0.22275 (18)	0.93481 (15)	0.0203 (3)	
H1A	−0.1228	0.2564	1.0402	0.030*	
H1B	−0.1567	0.1011	0.8914	0.030*	
H1C	−0.2906	0.2533	0.9016	0.030*	
C2	0.0631 (2)	0.23846 (16)	0.69410 (14)	0.0151 (3)	
C3	0.5248 (2)	0.66889 (17)	0.75182 (14)	0.0162 (3)	
C4	0.2779 (3)	0.89933 (17)	0.72402 (17)	0.0224 (3)	
H4A	0.1240	0.9264	0.6889	0.034*	
H4B	0.4291	0.9490	0.6872	0.034*	
H4C	0.3016	0.9447	0.8298	0.034*	

### Atomic displacement parameters (Å^2^)

**Table:**

	*U*^11^	*U*^22^	*U*^33^	*U*^12^	*U*^13^	*U*^23^
S1	0.01675 (17)	0.01802 (17)	0.01540 (17)	0.00122 (12)	0.00094 (12)	0.00507 (13)
S2	0.01809 (17)	0.01627 (17)	0.01596 (17)	0.00210 (12)	0.00280 (12)	0.00433 (13)
S3	0.01357 (16)	0.01617 (17)	0.02213 (18)	0.00337 (12)	0.00094 (13)	0.00725 (13)
S4	0.01479 (16)	0.01637 (17)	0.01887 (17)	0.00321 (12)	−0.00024 (12)	0.00678 (13)
O1	0.0178 (5)	0.0190 (5)	0.0212 (5)	0.0005 (4)	−0.0005 (4)	0.0048 (4)
O2	0.0210 (5)	0.0181 (5)	0.0297 (6)	0.0003 (4)	−0.0060 (4)	0.0070 (4)
C1	0.0189 (7)	0.0214 (7)	0.0213 (7)	0.0026 (5)	0.0049 (5)	0.0094 (6)
C2	0.0164 (6)	0.0130 (6)	0.0169 (6)	0.0058 (5)	0.0021 (5)	0.0050 (5)
C3	0.0151 (6)	0.0169 (6)	0.0184 (6)	0.0037 (5)	0.0031 (5)	0.0083 (5)
C4	0.0231 (7)	0.0162 (7)	0.0288 (8)	0.0053 (5)	0.0006 (6)	0.0090 (6)

### Geometric parameters (Å, º)

**Table:**

S1—C2	1.7553 (14)	O2—C3	1.2067 (17)
S1—C1	1.8057 (14)	C1—H1A	0.9800
S2—C2	1.8057 (13)	C1—H1B	0.9800
S2—S3	2.0332 (5)	C1—H1C	0.9800
S3—C3	1.8047 (14)	C4—H4A	0.9800
S4—C3	1.7528 (14)	C4—H4B	0.9800
S4—C4	1.8077 (14)	C4—H4C	0.9800
O1—C2	1.2037 (16)		
			
C2—S1—C1	98.04 (6)	O1—C2—S2	116.70 (10)
C2—S2—S3	105.23 (5)	S1—C2—S2	117.13 (7)
C3—S3—S2	105.85 (5)	O2—C3—S4	126.34 (11)
C3—S4—C4	97.96 (7)	O2—C3—S3	116.20 (10)
S1—C1—H1A	109.5	S4—C3—S3	117.46 (7)
S1—C1—H1B	109.5	S4—C4—H4A	109.5
H1A—C1—H1B	109.5	S4—C4—H4B	109.5
S1—C1—H1C	109.5	H4A—C4—H4B	109.5
H1A—C1—H1C	109.5	S4—C4—H4C	109.5
H1B—C1—H1C	109.5	H4A—C4—H4C	109.5
O1—C2—S1	126.15 (10)	H4B—C4—H4C	109.5
			
C2—S2—S3—C3	−90.99 (6)	C4—S4—C3—O2	1.13 (14)
C1—S1—C2—O1	1.09 (13)	C4—S4—C3—S3	−178.16 (8)
C1—S1—C2—S2	−177.21 (8)	S2—S3—C3—O2	−178.71 (10)
S3—S2—C2—O1	179.07 (9)	S2—S3—C3—S4	0.65 (8)
S3—S2—C2—S1	−2.46 (8)		
